# Human genetics seen through an evolutionary lens

**DOI:** 10.1016/j.xgen.2023.100323

**Published:** 2023-05-10

**Authors:** Chris P. Ponting

**Affiliations:** 1 MRC Human Genetics Unit, Institute of Genetics and Cancer, University of Edinburgh, Edinburgh EH4 2XU, UK

## Abstract

Most DNA bases crucial for species perpetuation are marked by a dearth of sequence change among species related over long evolutionary time. Recently, Christmas et al.^1^ and Sullivan et al.^2^ cast light on human DNA and its variants through comparison with 239 other mammalian species’ genomes.

## Main text

Human DNA, like any other genome, has been shaped by a strong survivorship bias. Bases of importance to species propagation have been retained preferentially, while others of less or no such importance have been mutated across tens or hundreds of millions of years of evolution. This explains why the degree of evolutionary conservation has long been used as a qualitative rule of thumb when assessing the importance of a gene or its regulatory elements. To provide a more detailed quantitation of evolutionary conservation, however, the genomes of 240 placental mammal species needed to be sequenced and multiply aligned.^3^ Species that are less closely related to humans, for example, birds or fish, were not chosen for this purpose despite providing the long evolutionary divergence needed to pinpoint highly conserved functional DNA. This is because mammalian phyla also carry DNA that has acquired functionality, or else has dispensed with it, only recently (∼10^7^ years) in a lineage-specific manner.^4^ Comparing human with bird or fish DNA would focus on deeply conserved functions but would often miss mammalian-specific innovations. In a recent paper, Christmas et al.^1^ further analyze these 240 genomes, finding that about 10% of the human genome is conserved across these mammals, about a third of which can be pinpointed at single-base resolution. This consortium’s second paper (Sullivan et al.^2^) then examined these conserved single bases, finding that variation at these sites occurs infrequently in our population, as expected from their deleteriousness.

Christmas et al. estimated that at least 11.6% (332 Mb of 2.85 Gb) of the human genome has been constrained over mammalian evolution. This is an important number to estimate because it is also the approximate fraction of all newly arising DNA variants that affect functional sequence. The estimate is a third higher than a previous prediction^4^ (253 Mb; 95% CI 220–286 Mb), which also took account of mammalian lineage-specific constraint. Christmas et al.’s estimates of constrained sequence varied widely across four other focal species (239–367 Mb), leading them to conclude that their method cannot determine whether the true extent of constraint varies among mammals. Conservation across mammals matched long-held notions of importance: protein-coding sequences, including their start and stop codons, were most enriched in conserved bases, followed by genes’ untranslated regions and promoters; also, developmental genes were most conserved, whereas environmental response genes were least conserved.

Impressively, the consortium could pinpoint a third of all constrained sequence (101 Mb) at single-base resolution. Half of this amount lies outside of the protein-coding sequence and is functionally unannotated, creating substantial opportunities for large-scale reverse genetics experiments. To gain insights into the molecular function of this vast genomic terra incognita, the Zoonomia team intersected a constrained sequence with 15.6 million transcription factor (TF) binding sites. The great majority (88%) of these sites, however, lacked conservation, and they found only a handful of TFs whose conserved binding sites outnumbered their non-conserved sites. This indicates that binding of TFs only rarely regulates transcription, as previously proposed.^5^ As Doolittle et al.^6^ suggested, only sites whose DNA variants are under selection should be deemed functional.

Sullivan et al.^2^ compared constraint that shaped mammalian genomes over a long evolutionary time with constraint applied across more recent human evolution. As expected, they found human genetic variation at mammal-constrained positions being preferentially purged from our population. Over a third of all human genes lacked any common or low-frequency variants at mammal-conserved sites, including many involved in fundamental processes such as in embryogenesis. Genes with greater human variation at mammal-conserved sites tended to be more involved in environmental response, including immunity. Human long non-coding RNA promoters and exons lacked mammal-conserved sites almost entirely, as reported previously.^7^ Overall, common and low-frequency human variants at mammalian-conserved sites were more likely to be pathogenic and to have a larger influence on human traits. These observations will help improve the fine-mapping of disease-causal variants and estimating the inherited liability of genetic disease.

Zoonomia has gifted science an important data resource whose value will steadily rise from future use. The project recapitulates past findings gleaned from much smaller datasets, principally that the less variable a DNA site is among humans, the stronger its constraint is across mammals. The project should also be viewed as signposting future genetics and genomics research opportunities. Seen with a critical eye, Zoonomia’s broad sampling of eutherian phyla will result in diminishing returns from future genome sequencing of additional species (the genomes of about 5,900 placental mammal species remain to be sequenced^1^). Also, their genome analyses make clear that few inferences can be drawn about each species’ distinctive traits from its genome sequence alone. Despite their claims,^1^ sadly it is unlikely that Zoonomia will substantively protect future biodiversity. Rather the project’s data may ultimately be viewed as cataloging the rich inheritance of a once widely successful animal clade.

Viewed more positively, future opportunities now present themselves. The first of these is a more accurate inference of constrained sequence. Zoonomia took advantage of methods that did not account for known mutation rate variation at the ∼10 Mb scale.^8^ Future methods that account for chromosomal location- and phyla-dependent variation may help pinpoint lineage-specific functional sequence, which is estimated to contribute about half of all constrained DNA in placental mammals.^4^ Second, Zoonomia alignments may reveal correlated evolutionary sequence changes in protein-coding or non-coding sequence, each indicative of multiple compensatory mutations and potentially revelatory of mechanism. Finally, Zoonomia has shone a bright light on more than 400,000 conserved non-coding elements (median size 20 nucleotides) that remain unannotated and unexplained. This re-emphasizes how incompletely regulatory function has been interrogated in cell-based assays to date. Particularly under-represented will be non-coding regulatory function in rare cell types or involved in early developmental processes or responding to environmental conditions difficult to replicate in laboratory conditions. The greatest challenge now is to couple Zoonomia’s deep evolutionary information with novel assays in order to reveal regulatory molecular mechanisms.
